# Supplementary material for: Resolving the full spectrum of human genome variation using Linked-Reads
Source: Genome Res. 2019 Apr;29(4):635–45. doi: 10.1101/gr.234443.118 (PMC6442396; doi:10.1101/gr.234443.118)
Supplement: Supplemental Material [file supp_gr.234443.118_Supplemental_longranger-2.2.zip › longranger-2.2.tar.bz2!/lr2.2-public/lib/stan/cmdstan/stan/doc/api/html/math_2matrix_2mdivide__right__tri__low_8hpp_source.html]

Stan: src/stan/math/matrix/mdivide\_right\_tri\_low.hpp Source File


|  |  |
| --- | --- |
|  | Stan  2.5.0  probability, sampling & optimization |


- Main Page
- Related Pages
- Namespaces
- Classes
- Files

- File List
- File Members

All Classes Namespaces Files Functions Variables Typedefs Enumerations Enumerator Friends Macros Pages

- src
- stan
- math
- matrix

mdivide\_right\_tri\_low.hpp

Go to the documentation of this file.

1 #ifndef STAN\_\_MATH\_\_MATRIX\_\_MDIVIDE\_RIGHT\_TRI\_LOW\_HPP

2 #define STAN\_\_MATH\_\_MATRIX\_\_MDIVIDE\_RIGHT\_TRI\_LOW\_HPP

3

4 #include <boost/math/tools/promotion.hpp>

5 #include <stan/math/matrix/Eigen.hpp>

6 #include <stan/math/matrix/mdivide\_right\_tri.hpp>

7 #include <stan/math/matrix/promote\_common.hpp>

8

9 namespace stan {

10  namespace math {

11

21  template <typename T1, typename T2, int R1,int C1,int R2,int C2>

22  inline

23  Eigen::Matrix<typename boost::math::tools::promote\_args<T1,T2>::type,R1,C2>

24  mdivide\_right\_tri\_low(const Eigen::Matrix<T1,R1,C1> &b,

25  const Eigen::Matrix<T2,R2,C2> &A) {

26  return mdivide\_right\_tri<Eigen::Lower>

27  (promote\_common<Eigen::Matrix<T1,R1,C1>,

28  Eigen::Matrix<T2,R1,C1> >(b),

29  promote\_common<Eigen::Matrix<T1,R2,C2>,

30  Eigen::Matrix<T2,R2,C2> >(A));

31  }

32

33  }

34 }

35 #endif

stan::math::mdivide\_right\_tri\_low

Eigen::Matrix< typename boost::math::tools::promote\_args< T1, T2 >::type, R1, C2 > mdivide\_right\_tri\_low(const Eigen::Matrix< T1, R1, C1 > &b, const Eigen::Matrix< T2, R2, C2 > &A)

Returns the solution of the system tri(A)x=b when tri(A) is a lower triangular view of the matrix A...

**Definition:** mdivide\_right\_tri\_low.hpp:24

promote\_common.hpp

mdivide\_right\_tri.hpp

Eigen.hpp

stan::math::promote\_common

common\_type< T1, T2 >::type promote\_common(const F &u)

**Definition:** promote\_common.hpp:14


---

[ Stan Home Page ]

*© 2011–2014,
Stan Development Team.*
